# The de novo transcriptome of the freshwater copepod *Cyclops abyssorum tatricus* reveals high-elevation adaptation

**DOI:** 10.1038/s41598-026-46084-x

**Published:** 2026-03-27

**Authors:** Ambre Placide, Morgan Kelly, Barbara Tartarotti

**Affiliations:** 1 Department of Ecology, University of Innsbruck, Technikerstrasse 25, 6020 Innsbruck, Austria; 2 Department of Biological Sciences, A309 Life Sciences Annex, Louisiana State University, Baton Rouge, LA 70803 USA

**Keywords:** Zooplankton, Long-read PacBio sequencing, Gene ontology, Alpine lakes, Ecology, Ecology, Zoology

## Abstract

Copepods, small aquatic crustaceans, are one of the most abundant zooplankton in the world. These animals play a critical ecological role in aquatic ecosystems such as oceans, streams, or, as in this study, alpine lakes. In these ecosystems, copepods have adapted to stressful and dynamic environments, a process which can be investigated via comparative transcriptomics. An assembled transcriptome is a pre-requisite to applying transcriptomic tools in physiological research, but there are few transcriptome assemblies available for copepods. To address this gap, we assembled a *de novo* transcriptome of *Cyclops abyssorum tatricus* by merging Pacific Bioscience long reads from copepods collected in two lakes at two different time points. The final assembly consisted of 52,521 contigs with a BUSCO score of 80.7%. We annotated a total of 26,255 (49.99%) protein sequences using the eggNOG database. Gene ontology analyses revealed that most gene annotations were involved in cellular processes and signaling (34.61%). Comparisons with two other copepod species showed that the transcriptome assembly of *C. abyssorum tatricus* is enriched for cold acclimation genes, consistent with its long-term adaptation to cold water environments. This *de novo* transcriptome will enable comparative transcriptomic studies in this species, allowing us to investigate physiological adaptations to alpine environments.

**Supplementary Information:**

The online version contains supplementary material available at 10.1038/s41598-026-46084-x.

## Introduction

In the aquatic food web of most lake habitats, zooplankton, like copepods, occupy an intermediate trophic position, feeding on microbes and acting as prey for a variety of invertebrates and vertebrates^1^. As they live in lakes with a wide range of physicochemical and optical characteristics, these species are subject to a range of environmental stressors. Copepods depend on phenotypic plasticity to adjust to this environmental variation by altering their biochemistry, physiology, behavior, or life history^2^ on short- or long-term timescales^3^. The biochemical and physiological processes underlying such plastic alterations may be linked to differential gene expression patterns, including RNA transcription, translation to polypeptides, and post-translational modification^4^. In harsh habitats like alpine lakes, vulnerable to environmental change^5^, plankton species must cope with oligotrophic conditions, low temperatures, and severe seasonal variations, thus, phenotypic adjustments are crucial. Recent studies have shown that copepods from alpine lakes rely on a variety of behavioral and physiological strategies to adapt to changes in environmental conditions^6^.

In the last decade, advances in high-throughput sequencing have enabled the time-efficient analysis of genomes and transcriptomes, and RNA sequencing (RNA-seq) has become a crucial tool in physiological research^7^. Comparative transcriptomic techniques have been successfully used to link physiological defense mechanisms to gene expression in copepods^8^, including stress-related genes^9^ and antioxidant enzymes^10^. Yet despite the time and cost efficiency of RNA-seq, these techniques have not been applied to zooplankton from freshwater lakes, and very rarely in cyclopoid copepods as their genomes are notoriously difficult to assemble^11^ due to low GC%, high repetitiveness and large genome size variability, complicating standardized assembly pipelines^12^.

In this study, we sequenced the transcriptome of the freshwater copepod *Cyclops abyssorum tatricus* and assessed the resulting assembly using RNA-seq. We sampled individuals across multiple lake types and time points to capture expression variation associated with adaptive processes (e.g., responses to cold and UV radiation). Using this assembly, we characterized this species’ transcriptome, describing genes related to biological processes and pathways according to their gene ontology (GO) terms. To investigate mechanisms underlying transcriptomic differentiation in alpine freshwater systems, we compared the *C. abyssorum tatricus* assembly with published transcriptomes from two marine copepods, the intertidal harpacticoid *Tigriopus californicus*^13^^14^ and the estuarine calanoid *Eurytemora carolleeae*^15^. Although these taxa differ in phylogenetic position, genome architecture, and sampling methods were not standardized across datasets, this comparison spans broad ecological and evolutionary diversity within the class Copepoda and provides context for a priori candidate gene categories associated with cold adaptation and high elevation.

This transcriptome assembly will be, to our knowledge, the first assembly of a purely freshwater copepod. It provides a transcriptomic baseline for future research on the molecular biology, evolutionary genomics, and adaptative physiology of freshwater copepods. This resource enables investigations into how copepods modulate gene expression to withstand environmental changes. It also facilitates differential gene expression analysis under different environmental conditions, helping identify key genes and pathways involved in cold adaptation, UV acclimatization or oxidative stress responses. This study not only improves our understanding of phenotypic plasticity at the molecular level but also contributes to a broader comprehension of how copepods, particularly freshwater cyclopoids, adapt, evolve, and persist in harsh environments.

## Results

### Sequencing statistics and quality control

Sequencing information is summarized in Supplementary Table S1. We generated approximately 12.9 million reads per sample. The raw sequencing data were PACBIO High quality HiFi reads^16^ with a 99.9% read accuracy and > 90% of bases Q30 + with uniform coverage. Initial sequencing of the raw FAS3 and FAS4 lake datasets generated BUSCO scores of 42.1% (with 19.4% of single copy and 22.6% of duplicated) and 47.6% (22.3% single copy and 25.3% duplicated), respectively for the odb10 datasets. The sequences were clustered using the Isoseq assembly pipeline and resulted in 767,735 read seq (5.55%) for FAS4 and 199,468 reads (0.64%) for FAS3.

After using the CDHIT pipeline to merge and cluster the two assemblies into one, our assembly contained 70,496 contigs (7.29% of the original read number). After running the CDHIT pipeline a second time to cluster similar proteins, we arrived at our final transcriptome assembly with 52,521 contigs described in Supplementary Tables S2 and S3. This assembly presents a BUSCO score to the Arthropoda of 80.7% with 34.5% single copy and 46.2% duplicated.

Transcoder identified 62,254 open reading frames in our assembly with a range of 87–2779 amino acids and an average and median length of 364.5 amino acids and 285.0 amino acids, respectively.

### Gene annotation

**Fig. 1:**
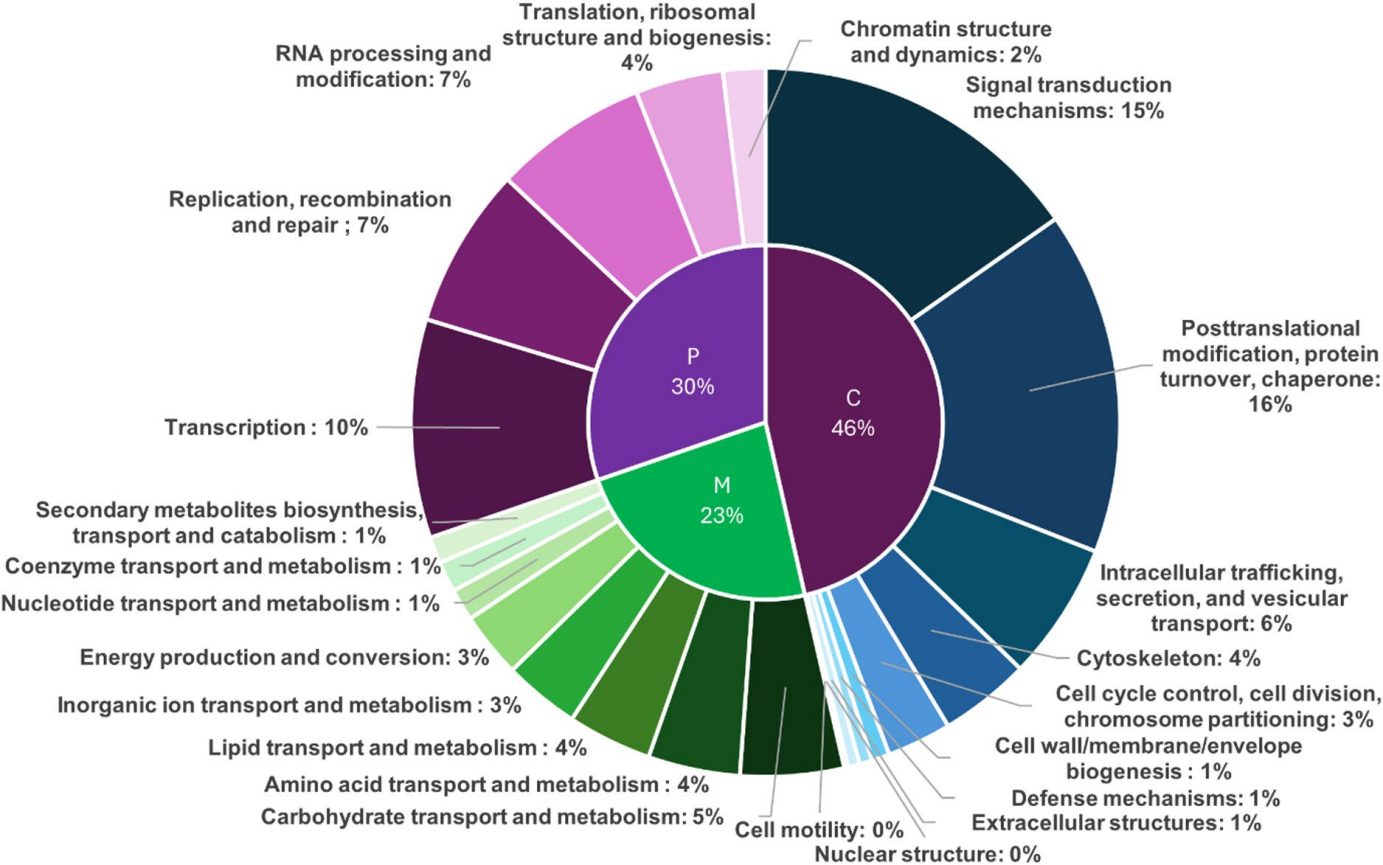
The 23 most abundant functional classes, grouped into three domains: cellular processes and signaling (**C**) (46%), information storage and processing (**P**) (30%) and metabolism (**M**) (23%), not included are the poorly characterized functions.

Gene annotation was performed in OmicsBox^17^ using DIAMOND to search the eggNOG blast database, with functional mapping carried out in Blast2GO^18^. Out of the 62,254 input sequences, 26,255 (42.17%) were successfully annotated, with a total of 335,108 GO annotations and an average of 12.76 annotations per sequence. The rarefaction curve (Supplementary Figure S1) approached an asymptote, showing the completeness of the assembly and indicating that additional searches would yield comparatively few new terms. Clusters of Orthologous Groups (COG) functional categories were assigned following NCBI’s Clusters of Orthologous Genes^19^. The classes were separated into four domains, with the most highly represented being the cellular processes and signaling (C) (31.21%), followed by the poorly characterized functions (22.90%), the information storage and processing (P) (20.35%), and metabolism (M) (15.65%). Figure 1 shows the 23 functional classes, not including the poorly characterized domains.

Approximately 10% of the annotated sequences were assigned to 13 GO terms: 4.4% in Cellular Component: nucleus (GO:0005634; 1.10%), cytosol (GO:0005829; 1.06%), cytoplasm (GO:0005737; 1.01%), nucleoplasm (GO:0005654; 0.62%), plasma membrane (GO:0005886; 0.61%); and 3.97% in Biological Processes: multi-organism reproductive process (GO:0044703; 1.33%), cellular anatomical entity morphogenesis (GO:0032989; 0.69%), positive regulation of gene expression (GO:0010628; 0.55%), regulation of biological quality (GO:0065008; 0.54%), localization of cell (GO:0051674; 0.45%), and negative regulation of gene expression (GO:0010629; 0.41%); and 1.79% in Molecular Function: heterocyclic compound binding (GO:1901363; 1.26%) and protein binding (GO:0005515; 0.53%).

The analysis of 335,108 GO terms using Blast2GO eggNOG-mapper revealed the presence of 12,788 unique GO terms, ranging from 1 to 4481 different GO terms in each of the 26,255 annotated proteins (Supplementary Figure S2). These were divided into 24 out of 25 available NCBI’s clusters of orthologous genes functional categories, with the general function prediction category being the only one missing. Cluster S (function unknown, *n* = 8542 genes) was the largest functional group (23.5%), followed by two clusters from the cellular processes and signaling domains. Clusters O (posttranslational modification, protein turnover, chaperone, *n* = 4420, 12.03%) and T (signal transduction mechanisms, *n* = 4305, 11.72%) were followed by two clusters from the information storage and processing domain, K (transcription, *n* = 2813, 7.66%) and L (replication, recombination and repair, *n* = 2066, 5.62%). Clusters N (cell mobility, *n* = 16, 0.04%) and Y (nuclear structure, *n* = 35, 0.10%) were the least represented.

### Homolog comparisons

With the goal of identifying ontology terms that are specific or enriched in the high-elevation freshwater cyclopoid copepod *C. abyssorum tatricus*, we compared our assembly with two other copepod species (*Tigriopus californicus* and *Eurytemora carolleeae*) with assembled and annotated transcriptomes.

### Orthologous gene cluster

We compared our 52,521 contig assembly with the 14,526 contig assembly of *E. carolleeae* and the 11,341 of *T. californicus*. This analysis identified 5889 ortholog clusters shared among all three species (Supplementary Figure S3), the next highest number of shared ortholog clusters were between *E. carolleeae* and *T. californicus* (2374), followed by *T. californicus* and *C.* *abyssorum tatricus* (923). This analysis was performed using the web-based genomics comparator OrthoVenn3^20^, employing the genome-scale protein clustering algorithm OrthoMCL^21^ with DIAMOND blastx similarity searches. A phylogenetic tree was constructed based on sequence identity of single-copy genes. Supplementary Figure S4 shows the relationship among the three species studied; the colored bars indicate the number of orthologous clusters for each species^22^.

### GO pathway comparison

**Fig. 2:**
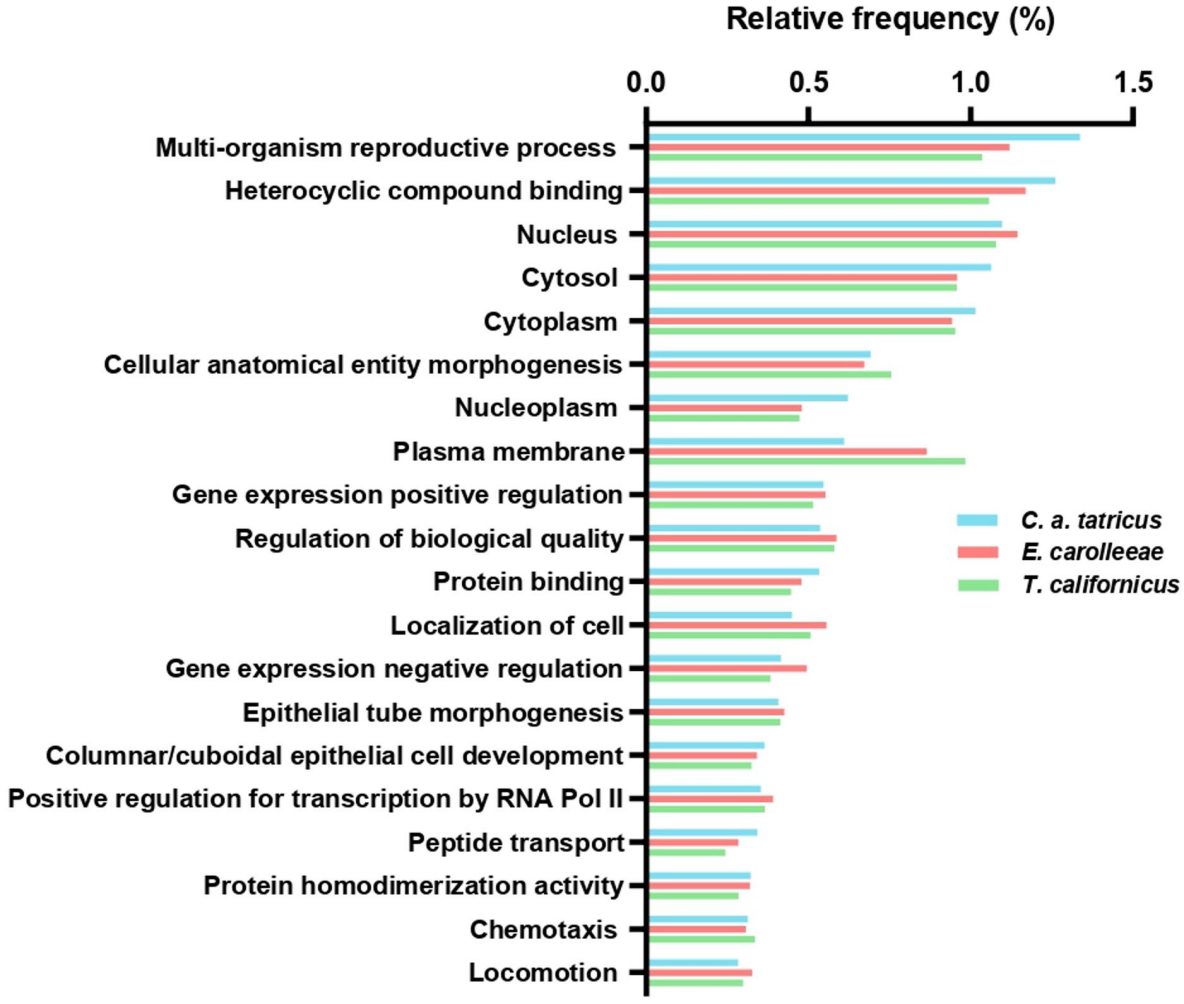
Relative frequency of the top 20 GO terms shared by all three copepod species.

We applied the same gene annotation pipeline to *E. carolleeae* and *T. californicus* as to *C. abyssorum tatricus*, using the available NCBI FASTA files. GO term frequencies were normalized within each species’ dataset. The most frequent GO term for *C. abyssorum tatricus* was multi-organism reproductive process (GO:0044703; *n* = 4481; 1.34%). For *E. carolleeae*, it was heterocyclic compound binding (GO:1901363; *n* = 2205; 1.17%), which ranked second in *C. abyssorum tatricus* (*n* = 4227; 1.26%). For *T. californicus*, the most frequent term was nucleus (GO:0005634; *n* = 1516; 1.08%), which ranked third in *C. abyssorum tatricus* (*n* = 3676; 1.10%) (Fig. 2).

## Discussion

This paper describes the first annotated transcriptome assembly of a freshwater cyclopoid copepod, the *C. abyssorum tatricus*. The number of contigs in this assembly (52,521) is within the range for other assemblies of calanoid copepods (38k-120k)^23^, and in the lower range of other cyclopoid copepod assemblies (32-125k)^12,24,25^. Both *C. abyssorum tatricus* samples had an RNA Integrity Number (RIN) below seven. Although this is acceptable and has been described as good in some studies^26^, it entails a risk of underperformance when isolating long sequence reads. This may have impacted the results of our assembly, as the RIN number is linked to the outcome of downstream experiments^27^. While their BUSCO scores (42.1% and 47.6%) are below the 80% threshold required for a BUSCO to be considered of good quality for eukaryotes, they are similar to that of the only cyclopoid copepod that has been analyzed using BUSCO^12^. Nevertheless, the transcriptome BUSCO score is 80.7%, which is within the 70–95% range observed in other copepods^14,23,28^. The relatively high proportion of duplicated complete copy (46.2%) suggests potential isoform redundancy or incomplete collapsing of transcripts between the two sets of raw data. Such duplication could influence future functional analyses. It may lead to the overrepresentation of certain gene families, causing the downstream gene ontology analysis to become artificially inflated. With a GC content of 40.85%, this species is comparable to another cyclopoid copepod (38.27 ± 4.06%^15^), but lower than the average for crustaceans (42.8–66.5%^29^) or copepods (36.08%±5.4%^12,15,30^). However, it was higher than that of 130 published marine invertebrate genomes^31^. Our N50 measurement (3111 pb) lies within the expected range for cyclopoid copepods (1680–4180 pb^12,24,25^), but is at the lower end of the range for copepods (1328 ± 2901 kb^30^) and marine invertebrates (418 ± 1241 kb^31^). Transcoder identified 62,254 putative open reading frames (ORFs) in the nucleotide sequences containing candidate coding regions, which is an expected result for copepods^23,24^.

The phylogenetic tree shows that, although *E. carolleeae* and *T. californicus* shared most clusters, *C. abyssorum tatricus* and *T. californicus* are more genetically similar. Morphologically based phylogenetic trees of the copepod orders show cyclopoids and harpacticoids to be derived relative to calanoids^32^. This inference is supported by their smaller adult transcriptomes and range in transcriptome size^33^.

**Fig. 3:**
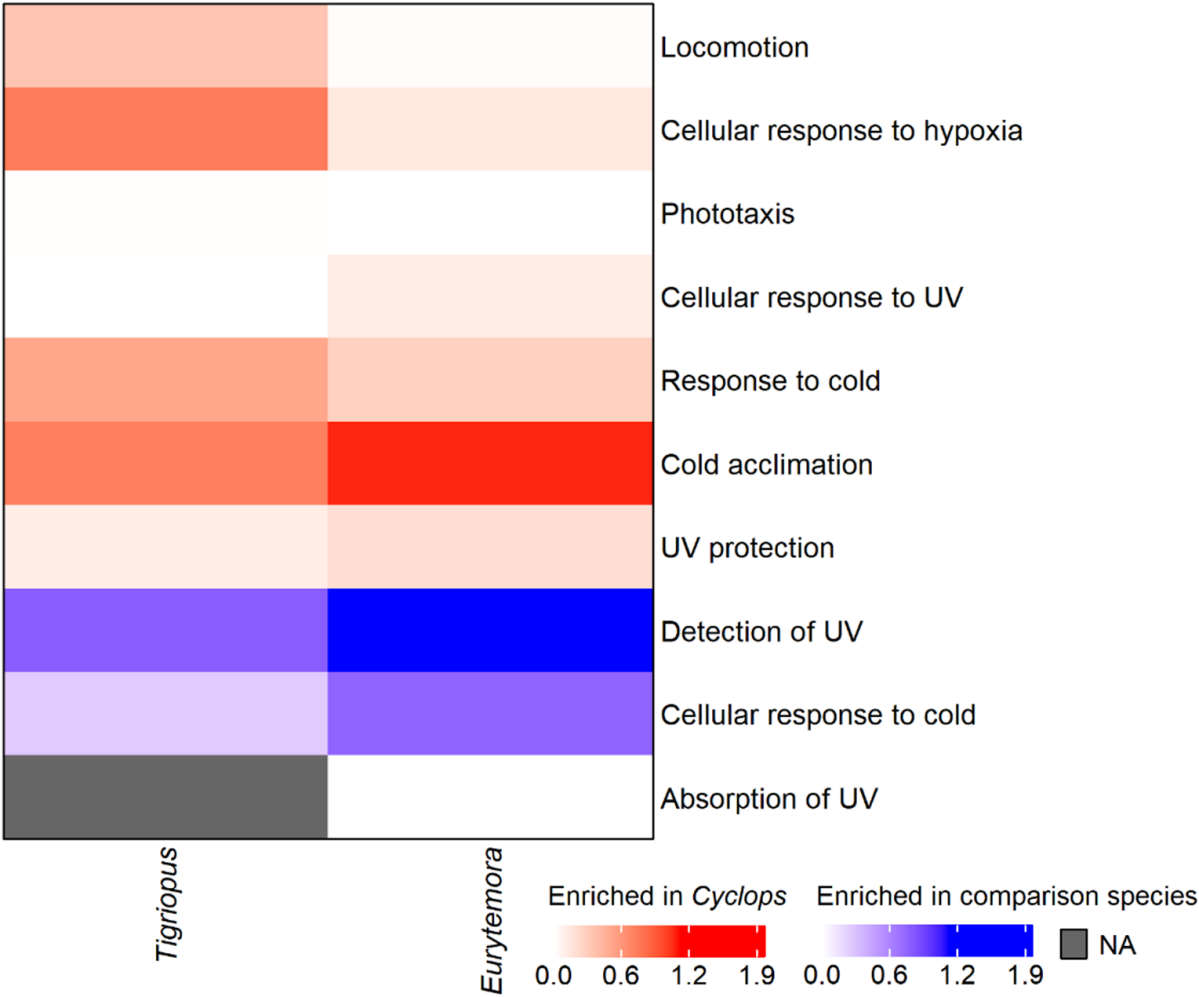
Heatmap of the Fisher’s exact test -log10(p-value) of *Cyclops abyssorum* *tatricus* compared to *Tigriopus californicus* (left side) and *Eurytemora carolleeae* (right side) for the ten GO terms of interest. Red indicates enrichment of GO terms in *C. abyssorum* *tatricus* relative to the other species. Blue indicates enrichment in the comparison species relative to *C. abyssoum* *tatricus*. “NA” (not available) denotes that the GO term is absent.

We identified twelve GO terms related to alpine lake environments (e.g., elevation and hypoxia). Of these, ten were detected in *E. carolleeae* and nine in *T. californicus* (Fig. 3). The two species shared seven GO terms. Although *C. abyssorum tatricus* exhibited higher enrichment for most terms, the overall overlap suggests that many of these functional categories are broadly conserved across copepods from diverse habitats.

Processes related to “Cold acclimation” and the “Response to cold” are strongly enriched in *C. abyssorum tatricus*, particularly relative to *E. carolleeae*. This suggests that *C. abyssorum tatricus* has a strong activation of cold-tolerance genes, consistent with long-term adaptation to cold environments. In contrast, the short-term stress response, which is characterized by the “Cellular response to cold”, is more prevalent in the other species, especially *E. carolleeae*. Seasonal differences in expression in the transcriptome assemblies of the two comparative species may contribute to these functional patterns.

The GO term “UV protection” is enriched in *C. abyssorum tatricus*, whereas the terms “Detection of UV” and “Absorption of UV” are enriched (particularly in *E. carolleeae*) or are comparable in the comparison species. This pattern suggests that *C. abyssorum tatricus* primarily emphasizes UV damage repair/protection, while *E. carolleeae* and *T. californicus* are more enriched for sensory or detection responses to UV radiation. For the term “Cellular response to UV”, no significant enrichment was detected, likely because this response is part of a general stress response across diverse habitats.

The behavioral terms “Phototaxis” and “Locomotion” show weak, nonsignificant enrichment across all three copepod species. This is not surprising given that vertical migration is common in copepods from both freshwater and marine systems^34,35^, reflecting shared ecological history rather than habitat-driven divergence. However, such behavior may be less ecologically relevant for *T. californicus*, which inhabits (very) shallow rock/tide pools. Our data further suggests that *C. abyssorum tatricus* does not exhibit freshwater-specific adaptations in light sensitivity or locomotor functions (e.g., feeding, swimming) relative to marine taxa.

The term “Cellular response to hypoxia”, potentially linked to prolonged ice cover in the habitat of *C. abyssorum tatricus*, is strongly enriched relative to *T. californicus* but only weakly enriched relative to *E. carolleeae*. Therefore, low oxygen adaptation seems not to distinguish *C. abyssorum tatricus* markedly from the marine copepods.

**Fig. 4:**
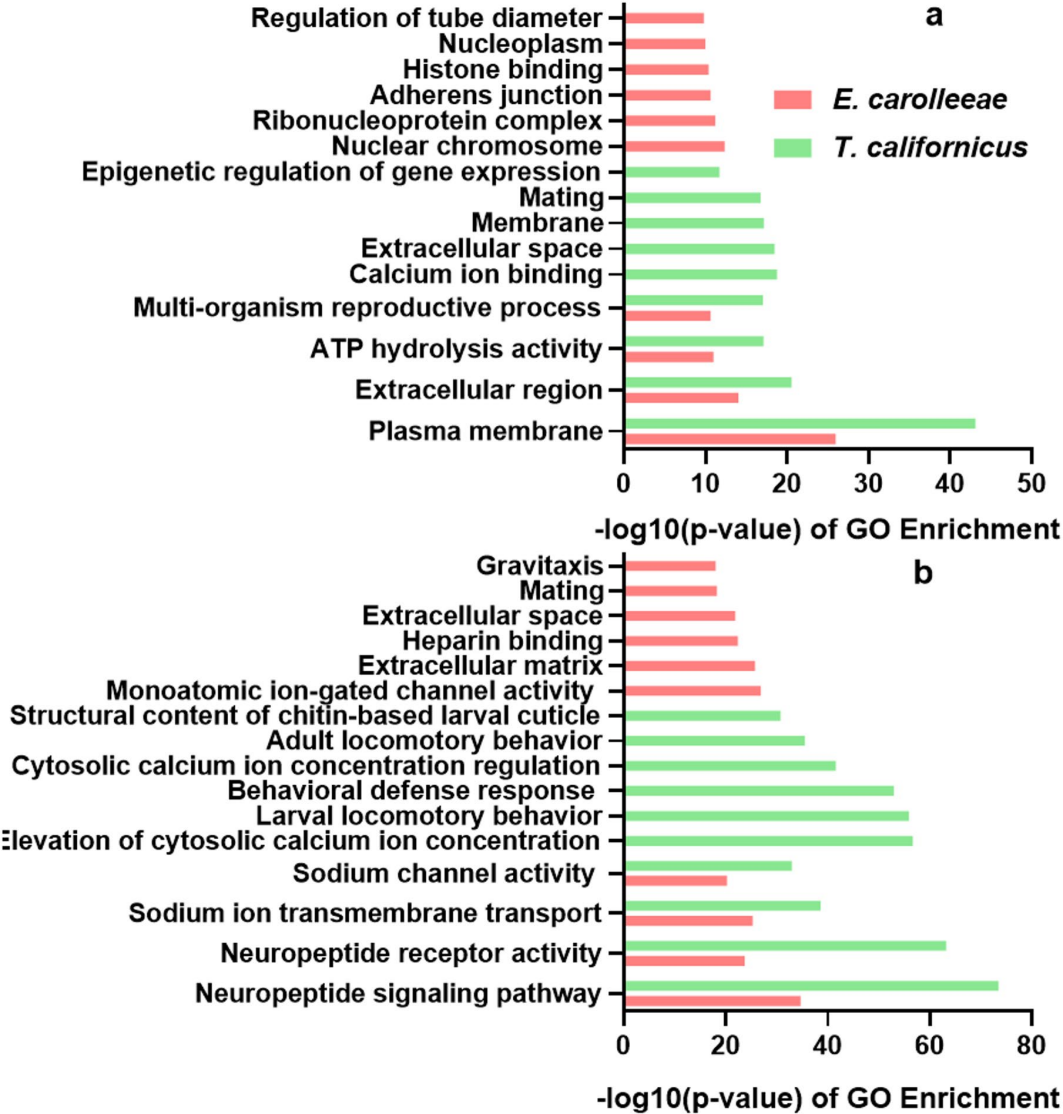
Top ten GO terms ranked by Fisher’s exact test -log10(p-value): (**a**) terms enriched in the *Cyclops abyssorum* *tatricus* assembly compared to the *Tigriopus californicus* and *Eurytemora carolleeae* assemblies; (**b**) terms enriched in *Tigriopus californicus* or *Eurytemora carolleeae* assemblies relative to *Cyclops abyssorum* *tatricus*.

We also assessed the GO terms with the strongest enrichment (i.e., highest Fisher’s exact test -log10(p-value)) in *C. abyssorum tatricus* compared to the other two copepod species, and in *E. carolleeae* and *T. californicus* relative to our species of interest (Fig. 4).

Distinct functional patterns emerge among the copepod species. In *C. abyssorum tatricus*, GO terms linked to gene expression regulation and cellular organization, such as epigenetic regulation of gene expression, ribonucleoprotein complexes, adherens’ junctions, and nucleoplasm, are enriched. By contrast, *T. californicus* shows an enrichment for terms associated with cellular communication and ion regulation (membrane, extracellular region and calcium ion binding) compared to *C. abyssorum tatricus*.

Contrarily, genes enriched in *T. californicus* and *E. carolleeae* are associated with neural, sensory and behavioral functions. In *T. californicus*, genes involved in locomotory behavior or behavioral defense responses in adults or larvae are significantly enriched, indicating that molecular processes related to movement or mobility are more transcriptionally active and important than in *C. abyssorum tatricus.* Both *T. californicus* and *E. carolleeae* also show strong enrichment for sodium-related GO terms characteristic of marine habitats^9,15^.

Because the enrichment analyses rely on Fisher’s exact tests of relative GO term representation, the results should be viewed as indicative trends rather than definitive evidence of long-term adaptive evolution. Even so, comparing differences in GO representation can provide useful insights into the distribution of functional capacities among copepods from diverse habitats. These findings provide a framework for identifying candidate categories for follow-up via differential gene expression analyses or experimental approaches.

## Materials and methods

### Sampling

Specimens of *Cyclops abyssorum tatricus* (Kozminski, 1927), a species commonly found in lakes of the Eastern Alps and other alpine regions^6^, were sampled from two alpine lakes situated above the tree line in the Austrian Central Alps, approximately 200 m apart. Faselfadsee 3 (FAS3) is located at an elevation of 2416 m above sea level (a.s.l.) and has a maximum depth of 17 m. It is still under glacial influence, which causes turbidity of ~ 4 NTU in summer. Faselfadsee 4 (FAS4), located at an elevation of 2420 m a.s.l., has a maximum depth of 15 m and is not connected to the glacier, making it a clear lake. Samples were collected at two different times, 14 July 2023 for FAS4 during the ice-free period and on 7 December 2023 for FAS3 when the lake was ice-covered. Samples were taken by vertical net tows using a plankton net (50 μm mesh size) around midday on sunny days at the deepest point of the lakes. For each sampling date, four replicates of 60 to 100 copepods (including all life stages; mostly copepodid stages) were picked immediately after sampling, placed in 2 ml Eppendorf tubes, flash-frozen, and stored at − 80 °C. The copepod populations consist of genetically identical species (based on the mitochondrial cytochrome c oxidase subunit I gene; P. Kirschner, M. Ventura, R. Sommaruga, unpubl.).

### Workflow

**Fig. 5:**
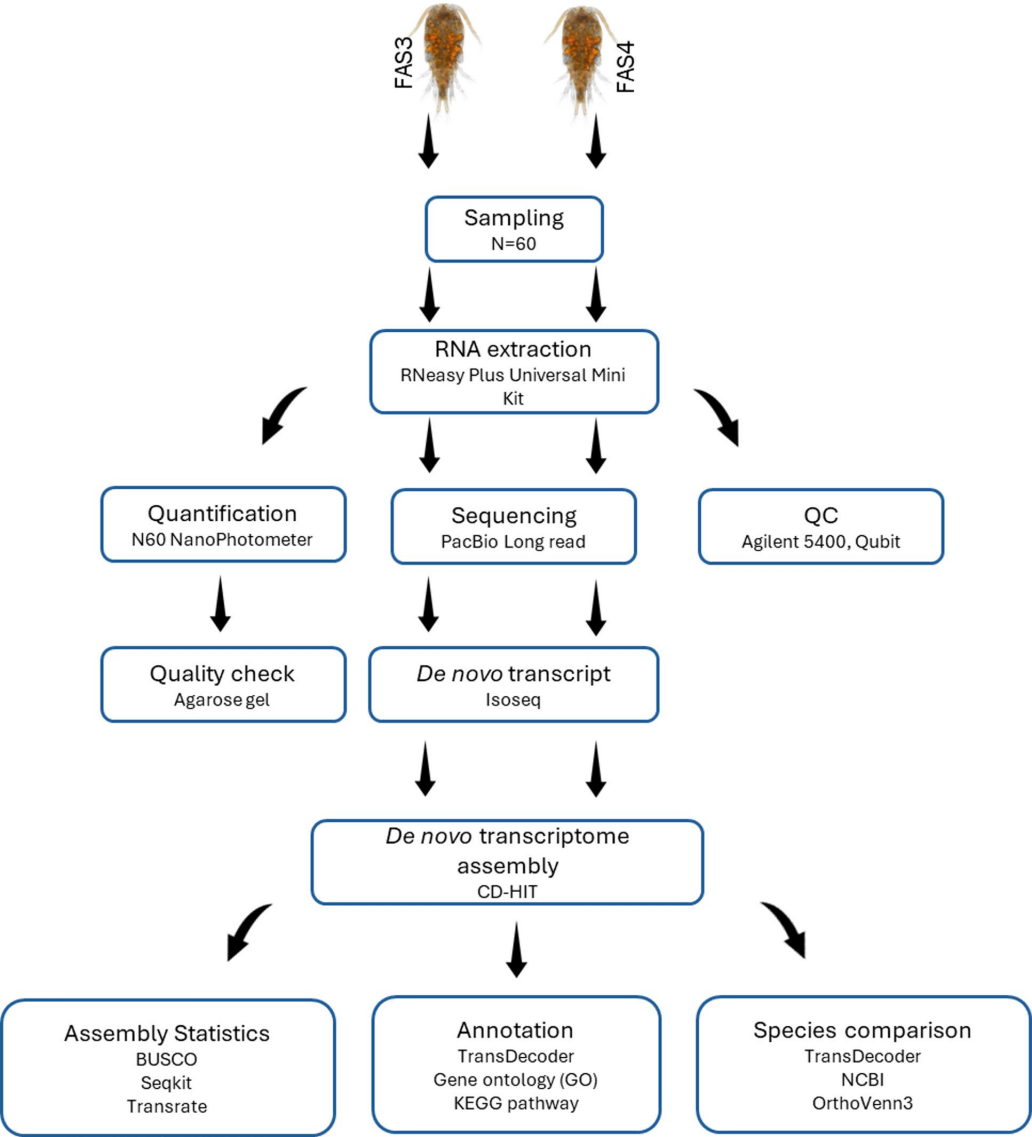
Workflow from the sampling to the transcriptome assembly.

A diagram of the workflow used in this study is presented in Fig. 5, and each step will then be described.

### RNA isolation, quantification and quality control

RNA was extracted as previously described in detail^36^. Briefly, we used TRIzol reagent (Invitrogen) to extract total copepod RNA, following the manufacturer’s instructions. The samples were homogenized and gDNA eliminator solution (Qiagen) was added to eliminate potential genomic DNA contamination. The RNA was then purified further using the RNeasy Plus Universal Mini Kit (QIAGEN, Germany), according to the manufacturer’s protocol, but using RW1 wash buffer instead of the supplied RWT buffer. The quantity and quality of the total RNA were determined by measuring 2 µL of the sample in an N60 NanoPhotometer (Implen, Germany). Samples were measured in duplicate (technical replicates). For long-read sequencing, 21 µL of the highest quality sample (mean A260/280 ratio: 2.203) of the four replicates for each lake were used. Additionally, a quality check was performed using a 1.2% agarose gel with GelRed (ThermoFisher Scientific).

### Sequencing

Sequencing was performed by Novogene Co., Ltd. Long-read sequencing was performed using a Pacific Biosciences (PacBio) HiFi library, which produces highly accurate cDNA long reads (HiFi reads) to characterize the entire assembly using Sequel IIe equipment for single-molecule real-time sequencing^16^ after quality control (Agilent 5400) and RNA quantification (Qubit).

### Processing and assembly

Raw long-read sequence data was processed using a shortened Isoseq v3^37^ workflow to generate transcripts by clustering HiFi reads on Conda v23.11.0 on Linux 64 v4.18.0. For every sequencing run, only the first three steps of the workflow were used. In short, the first step is to provide a single representative circular consensus sequence (CCS). The second step involves removing the primers provided by PacBio, and the third step consists of refining the dataset by removing poly(A) tails and identifying concatemers. The two nucleotide datasets were merged and clustered with CD-HIT v4.8.1^38,39^ and clustered a second time to cluster similar sequences into threshold (0.95) similar cluster reducing significantly the size of the databases by up to 1600 times.

### Assembly quality control

To validate the assembly process, we performed multiple post-assembly quality control checks^40^. Firstly, we assessed the sequence length statistics, such as the average, maximum and minimum, as well as fragmentation and simple statistics, including Q%20, GC%, and N50, using SeqKit v2.8.2^41^.

We used the tool BUSCO (Benchmarking Universal Single-Copy Orthologs) v5.8.3^42^ in euk_tran mode with zhr decency hmmsearch v3,1, MetaEuk v7, bba0d80 and Python 3.8.15 to test our assembly for orthologues of specific genes that are universally expressed and nearly exclusively found in the genome as single copies. The assembly’s completeness is determined by how many of the universal genes have matches in the input data (here arthropoda_odb10^43^ lineage dataset; 1013 single-copy orthologs; accessed January 2025), as well as whether these matches are full length, duplicated, or fragmented. A high-quality assembly should have a relatively high BUSCO score, the closest to 100% and very few missing or fragmented matches. Finally, we BLAST-aligned the contigs against the online NCBI^44^ non-redundant (nr) protein database (downloaded 2025-01-14) to assess sequence similarity to other species with BLASTX, using an E-value cutoff of 1e−05.

### Functional annotation

To produce a full transcriptome annotation, the assembly was first translated to amino acid sequences that code for predicted proteins, then translated into a protein prediction sequence using TransDecoder v5.7.1^45^, with only the best open reading frames resulting in full-length proteins of at least 100 amino acids. The gene ontology annotation was then realized using the eggNOG mapper v2.1.12^46^ workflow. In short, we compared our assembly with precomputed orthologous groups (OGs) and phylogenies from the DIAMOND eggNOG database v5.0.2, using Blast2GO eggNOG-Mapper 2.1.0^47^ to transfer their functional annotations. For GO enrichment, the target ortholog set was tested against a background of all predicted ORFs. Ortholog/GO assignments were accepted at an E-value cutoff of 1e−3, and only GO terms with non-electronic evidence were retained. Protein sequences were exported in FASTA (.pep) format for downstream analyses. Twelve specific GO terms related to either elevation (hypoxia), UV acclimatation^48^, swimming behavior or cold responses^49^ were selected: cold acclimation (GO:0009631), detection of UV (GO:0009589), absorption of UV (GO:0016039), response to cold (GO:0009409), locomotion (GO:0040011), cellular response to cold (GO:0070417), response to UV (GO:0009411), UV protection (GO:0009650), cellular response to UV (GO:0034644), cellular response to hypoxia (GO:0071456), response to hypobaric hypoxia (GO:1990910), and phototaxis (GO:0042331), using the EMBL’s European Bioinformatics Institute database. These all belong to the information storage and processing (P) term. To identify putative specialization to the alpine freshwater environment, we compared the *C. abyssorum tatricus* transcriptome to two coastal copepods, *E. carolleeae* (an estuary species) and *T. californicus* (a tidepool species). Reference transcriptomes were obtained from NCBI: *T. californicus* RefSeq GCF_007210705.1 (file: Tcal_SD_v2.1_rna.fna.gz); PacBio RSII; mixed lab-reared copepodids; 2019 release) and *E. carolleeae* RefSeq GCF_000591075.1 (file: Eaff_2.0_rna.fna; PacBio long-read; egg sample; 2017 release), both the most recently updated transcriptome assemblies available at the time of the study. The RNA FASTA files were downloaded and re-annotated using the same pipeline applied to *C. abyssorum tatricus* (including ORF prediction and protein/GO assignment), thereby generating harmonized annotations for this study. GO term abundances were normalized by the total number of annotated genes per species to account for assembly/annotation size, and enrichment was tested using the Fisher’s exact test with Benjamini-Hochberg correction (significance at FDR < 0.05).

## Supplementary Information

Below is the link to the electronic supplementary material.

Supplementary Material 1

## Data Availability

All elements of this study are available to the general public via the NCBI BioProject PRJNA1377012 database (https://www.ncbi.nlm.nih.gov/bioproject/PRJNA1377012/). This unannotated transcriptome shotgun assembly project has been deposited at DDBJ/EMBL/GenBank under the accession number GLLG00000000. The version described in this paper is the first version, GLLG01000000, while the two raw PacBio datasets are accessible via the accession numbers SAMN53755002 (run number: SRR36346890 in .bam or SRR37062719 in .fastq) and SAMN53755001 (run number: SRR36346891 in .bam or SRR37062720 in .fastq). No custom code has been used in the creation of the dataset for this article.
