# Supplementary material for: The de novo transcriptome of the freshwater copepod Cyclops abyssorum tatricus reveals high-elevation adaptation
Source: Sci Rep. 2026 Mar 27;16:10945. doi: 10.1038/s41598-026-46084-x (PMC13039363; doi:10.1038/s41598-026-46084-x)
Supplement: Supplementary file 1 — Supplementary Material 1 [file 41598_2026_46084_MOESM1_ESM.pdf]

Supplementary information

Table S1: Sequencing statistics for two PacBio long-read raw datasets from the copepod Cyclops abyssorum tatricus (sampled from lakes FAS3 and FAS4), including SeqKit metrics and BUSCO summaries. All lengths are reported in kb.

Lake	*BUSCO arthropoda_ odb10 (%)*	*RIN*	*Number of reads*	*Min length*	*Mean length*	*Max length*	*Q1 (bp)*	*Q2 (bp)*	*Q3 (bp)*	*N50 (bp)*	*GC (%)*
FAS3	42.1	6.6	12,120,462	52	2,656	303,429	1,797	2,436	3,112	2,833	42.82
FAS4	47.6	6.5	13,826,083	54	2,567	298,498	1,757	2,384	3,023	2,782	42.16

Table S2: De novo assembly sequence statistics for the copepod Cyclops abyssorum tatricus reported by SeqKit. All lengths are reported in kb.

	Min Length (nb sequences)	*Mean Length (nb sequences)*	*Max Length (nb sequences)*	*Q1 (nb sequences)*	*Q2 (nb sequences)*	*Q3 (nb sequences)*	*N50 (bp)*	*GC (%)*
De Novo	66	2,896	10,992	2,164	2,755	3,464	3,111	40.85

Table S3: BUSCO completeness statistics for the Cyclops abyssorum tatricus de novo assembly (1,013 ortholog groups searched)

	BUSCO arthropoda_odb10	*BUSCO eukaryote_odb10*	*BUSCO crustacea_ odb10*	*Complete seq (nb sequences)*	*Complete and single-copy (nb sequences)*	*Complete and duplicate (nb sequences)*	*Fragmented (nb sequences)*	*Missing (nb sequences)*
De Novo	80.7%	79.8%	76.2%	819	244	575	31	163

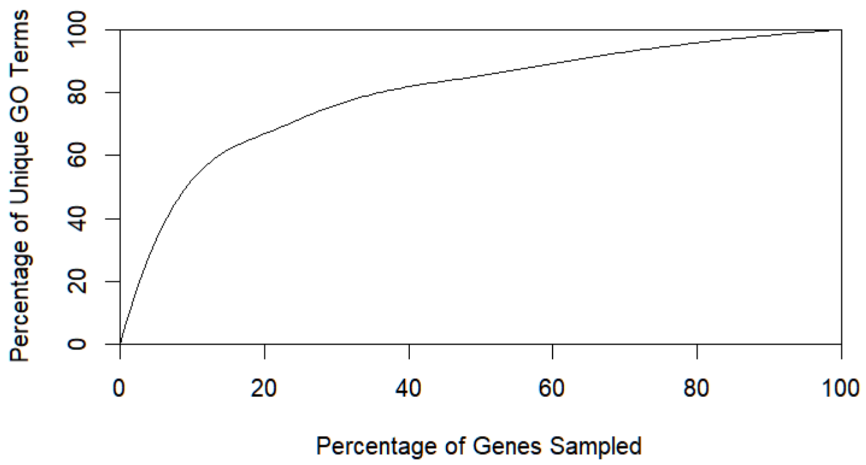

Figure S1: Rarefaction curve of Gene Ontology (GO) annotation completeness for the Cyclops abyssorum tatricus assembly.

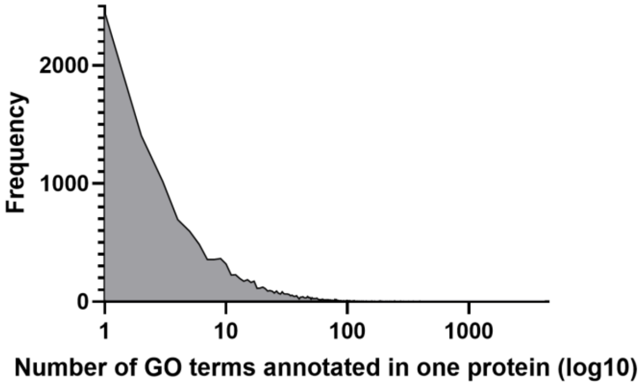

Figure S2: Distribution of the number of unique GO terms assigned per annotated protein (n=26,255) on a log_{10} scale.

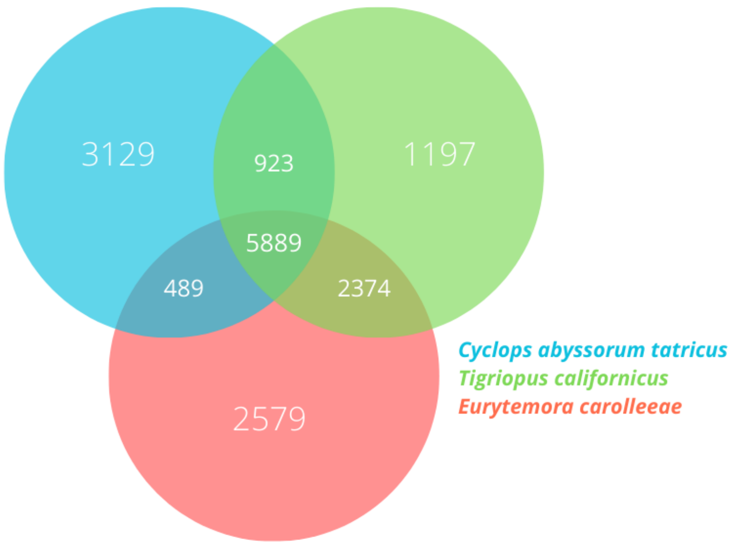

Figure S3: Venn diagram of ortholog clusters shared among Cyclops abyssorum tatricus, Tigriopus californicus and Eurytemora carolleeae. Homolog clusters were generated based on sequence similarity in OrthoVenn3.

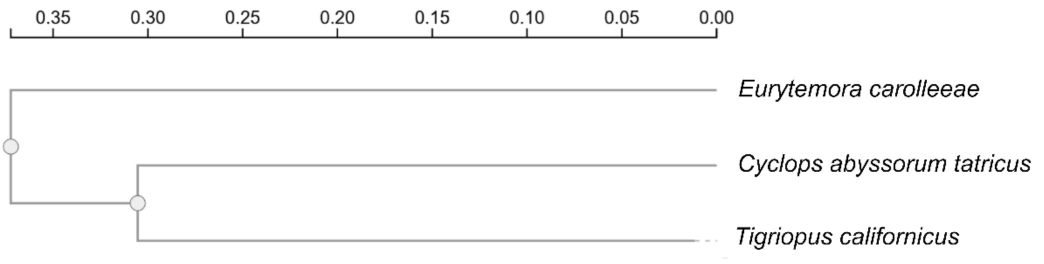

Figure S4: Phylogenetic tree showing relationships among Cyclops abyssorum tatricus, Tigriopus californicus and Eurytemora carolleeae using the maximum likelihood method, with reliability assessed via the Shimodaira-Hasegawa (SH) test; the scale bar indicates the expected number of substitutions per site.
